# Does Job Burnout Mediate Negative Effects of Job Demands on Quality of Life among Social Workers in Serbia?

**Published:** 2019-04

**Authors:** Ivana LJILJANA ZUBIC

**Affiliations:** Department of Psychology, Faculty of Philosophy, University of Niš, Niš, Serbia

## Dear Editor-in-Chief

Social workers are at a higher risk of work-related stress, burnout, and a lower quality of life compared to the general population and other health professionals due to high demands of their services and functioning in emotionally intense environments with limited resources. The Job Demands-Resourcesexplanatory model of burn-out was the theoretical framework of the study. The JD-R model (1) assumes that all occupations have burnout risk factors categorized as either a job demand or a job resource. Job demands and resources instigate two very different processes: 1. an energetic process of wearing out in which high job demands exhaust employees’ mental and physical resources and may, therefore, lead to burnout, and eventually to ill health; and 2. a motivational process in which job resources foster work engagement which in turn leads to positive organizational outcomes.

The aim of the study was to investigate an energetic process by examination of mediating role of job burnout in the relationship between job demands (workload, emotional demands) and quality of life in the group of social workers in Serbia. Six social services departments from different cities in Serbia were approached from May to November in 2016. Having obtained permission from the six respective Directors of Social Services, team leaders and social workers were asked for co-operation in filling in the research questionnaire. In some cases, by request, the researcher attended staff meetings to administer the questionnaire. In other cases, respondents were handed the questionnaire (and a covering letter) by their team leader and asked to return it, completed, in a sealed envelope direct to the researchers or to an agreed collection point. The confidentiality of the answers was emphasized.

The sample population consisted of 102 social workers working in Serbia (16 men and 86 women, average age of the sample 38.73 (SD=10.67)). In this study were used questionnaires for measuring workload (2), emotional demands (3), job burnout (Copenhagen Burnout Inventory), and quality of life (4). Mediation was tested using Preacher and Hayes Mediation test (5).

The findings proved the full mediation of burn-out in the relationship between workload and quality of life(R=0.34, *P*=0.00). Results showed a positive association of workload and burnout (β=0.52, *P*=0.00), a negative association between burnout and quality of life (β= −0.44, *P*=0.00) and negative association between workload and quality of life (β= −.33, *P*=0.00). Workload was no longer a significant predictor of quality of life after controlling for the mediator-burnout (β= −0.1046, *P*=0.1941), consistent with full mediation. The bootstrapping results (approach with 5000 samples) revealed that there was a significant negative indirect association between workload and quality of life through burnout (BCACI = 0.36 to 0.12; 95 % CI). The mediation test proved that burnout fully mediates the link between emotional demands and quality of life (R=0.34, *P*=0.00). Results indicated a positive association of emotional demands and burnout (β=0.7242, *P*=0.00), negative association between burnout and quality of life (β= −.4340, *P*=0.00), a negative association between emotional demands and quality of life (β −.4572, *P*=0.00). Emotional demands were no longer a significant predictor of quality of life after controlling for the mediator-burnout (β = −.1429,*P*=.1887), consistent with full mediation. The bootstrapping results (approach with 5000 samples) revealed that there was a significant negative indirect association between emotional demands and quality of life through burnout (BCACI=−.4990 to −.1575; 95% CI).

The results confirmed the existence of energetic process in which burnout mediated the effect of job demands (workload, emotional demands) on quality of life. This study verified the JD-R model of burnout among social workers in Serbian conditions. Study results can help inform management strategies for the prevention of job burnout development among social workers. Organizations of social work should aim to decrease job demands when feasible. Since burnout plays a key role in the model, individual-based interventions to reduce burnout symptoms might also be an avenue to explore.
